# The impact of mediated learning on the academic writing performance of medical students in flipped and traditional classrooms: scaffolding techniques

**DOI:** 10.1186/s41039-021-00165-9

**Published:** 2021-06-30

**Authors:** Laleh Khojasteh, Seyyed Ali Hosseini, Elham Nasiri

**Affiliations:** 1 Department of English Language, School of Paramedical Sciences, Shiraz University of Medical Sciences, Shiraz, Iran; 2 School of Nursing, Larestan University of Medical Sciences, Lar, Iran

**Keywords:** Academic writing, Flipped classroom, Mediated learning, Scaffolding

## Abstract

Writing as a multiple-step process is one of the most complex and demanding skills for graduate students to master. Foreign or second language learners who are required to write for academic purposes at the university level may even find it more demanding to master. One of the ways of decreasing the burden of mastering this skill for learners is mediation, using scaffolding techniques to teach writing. Hence, having a good understanding of the impact(s) of adopting mediating or scaffolding techniques in writing classes is absolutely indispensable. To this end, the present study employed an experimental research design to investigate the impact of mediation in the flipped writing classrooms of the students of medicine. To peruse this goal, 47 medical students were selected through purposive sampling and put into control and treatment groups. Medical students in the treatment group watched teacher-made video content(s) before their writing classes. The students in this group experienced organized-interactive writing group activities in their classes. Unlike the experimental group, the students in the control group received all the instructions in the classroom and were assigned homework. The findings obtained through the ANOVA and t-test indicated that the students in the experimental group significantly outperformed their counterparts in the control group in terms of their writing. A probable conclusion could be that by requiring students to study in advance and take responsibility for their learning, flipped classroom can provide the opportunity for learners to actively construct knowledge rather than receive the information passively in the classroom. Flipped classroom can also cultivate interactive class time for teachers and enable them to invest in more fruitful academic practices, instead of asking students to spend a substantial amount of time each week doing homework independently.

## Introduction

Scholarly writing presented in reports, essays, dissertations, and journal articles is a necessity for interpersonal communication in the scientific community. Lack of expertise or unfamiliarity with the principles of academic writing can seriously harm mutual understanding and hinder the smooth transition of the intended message (Kafipour, Mahmoudi, & Khojasteh, 2018).

Writing as a multiple-step process is one of the most complex and demanding skills for graduate students to master, particularly for L2 learners who need to write for academic purposes at the university level (Barroga & Mitoma, 2019). These students need to learn not only how to write in English, but also how to apply academic terminology, writing conventions, and higher-order thinking skills if they intend to have a voice in the disciplinary communities. Among other things, the students’ inability to master writing and the problems associated with it can be attributed to the lack of an explicit and systematic writing instruction for graduate students (Harris, 2006). Academic writing poses a daunting challenge to students because the rules of the “game” are almost all implicit (Casanave, 2002, p.19). Not only students but writing instructors teaching mixed-ability classes face the same dilemma of motivating all students to write well in English across diverse settings and for diverse audiences.

Mediation, as a scaffolding literacy process, is considered as one of the ways through which students, previously challenged with academic writing, can produce high-quality work and present their findings in scientific discourse (Rose, 2002). Closely related to the concept of mediation is scaffolding, which originated from the work of Wood, Bruner, and Ross (1976) who used this metaphor to describe a situation in which a teacher assists their students to master certain skills until they become capable of performing them independently.

The skilful gradual withdrawal of the support or scaffold is the common key feature in scaffolding techniques if the responsibility is to be successfully transferred from the veteran (teacher) to the novice (learner(s)). Based on the kind of support given to the novice, scaffolding can be divided into hard and soft types, (Saye & Brush, 2002). In the former, which is dynamic, situation-specific support is provided by the veteran during the learning process. In the latter, which is static, scaffolds are pre-planned and tailored based on the learners’ specific needs. This implies that the main difference between the two types of scaffolding is planning and the timing of the scaffolds provided.

Taking into account both the support quality and the time allocated to independent working, it is often argued that scaffolding, particularly the hard type, is very time-consuming. Since scaffolding is rather a process than a product, it requires substantial time and effort to adequately model the desired sequential series of strategies and pre-planned activities that are gradually reduced by the instructor when the appropriate time comes. Unfortunately, allocating this amount of time for deep learning at the university level with its jam-packed syllabi is almost impossible.

One of the challenges that confront writing instructors at Shiraz University of Medical Sciences is that the writing syllabus is often too packed to enable writing instructors to appropriately apply the mediation the medical students need. Therefore, lack of ability in generating, developing, clarifying, and organizing their ideas in an academically persuasive manner is evident in medical students’ writing outputs. Some studies have attributed this problem to the lack of explicit and systematic instruction in academic writing, lack of motivation, and lack of purpose in writing in the target language (i.e., Khojasteh, Shokrpour, & Kafipour, 2018). Moreover, the majority of medical students in Iran, particularly during the first 2 years of their study, do not have time or inclination to put more effort into their ESP classes.

Flipped classroom can be an alternative to the problem of the time constraint in writing classes. Although flipped classroom does not necessarily involve technology (i.e., Cheng, Ritzhaupt, & Antonenko, 2018; Hew & Lo, 2018), in the present study, a flipped classroom is defined as one in which instructions are delivered in a digital format. In such classes, instructor-made videos are viewed by the students outside the class, and the class time is devoted to organized interactive group activities. With the spread of the Covid-19 pandemic and the restrictions imposed on education, the significance of flipped classroom during the pandemic is even more pronounced. At the same time, the effectiveness of adopting such approaches needs to be scrutinized. The present study was therefore conducted to assess the impact of flipped classrooms on the ESP medical student writing performance.

### Literature review

Flipped classroom originated from the work of Jonathan Bergmann and Aaron Sams who used pre-recorded chemistry lectures to provide instruction to their students who were missing classes. The teachers were also interested in freeing up class time and devoting it to more fruitful activities that enabled them to consider the subject matter (chemistry) more comprehensively (Bergmann & Sams, 2012). Reportedly, flipped classroom boosts motivation and promotes autonomy as well as achievement in students because it not only provides opportunities for students to feel experienced in their ability, but also causes them to feel connected to their peers and teachers during the period of learning (see Hung, 2015; Murray, Koziniec, & McGill, 2015; Yang, Yin, & Wang, 2018).

Although, reportedly, teachers might face pedagogical, cognitive, and ergonomic challenges while implementing technology in their classes, students generally gain a lot when innovative educational techniques are utilized by teachers (Eshet, 2004, 2007; Koehler & Mishra, 2009). Zheng, Chu, Wu, and Gou (2018), for example, reported that the combination of offline and flipped classroom activities enhanced students’ learning outcomes and achievements. This is supported by another study carried out by AlJaser (2017) who reported that implementing flipped classroom made learning more productive for her students and made teaching and lecturing more interesting to her. A plethora of other studies have reported similar findings (i.e., Al-Zahrani, 2015; Basal, 2015; Elian & Hamaidi, 2018; Evseeva & Solozhenko, 2015 to name a few). However, studies addressing the implementation of flipped classroom for teaching academic writing at higher education are scarce. The present study therefore intends to address flipped classroom with respect to ESP writing classes. It was assumed that flipping the classes would create more room for implementing specific scaffolding techniques such as modelling, bridging, contextualization, schema-building, and text representation. A further assumption was that mediation would motivate or increase the motivation of demotivated or less motivated medical students and increase the chances of guided academic writing instruction.

The present study intended to investigate whether mediation in flipped writing classroom is more effective than conventional classrooms in improving the students’ writing performance. The writing performance of students in the control group and experimental group was compared to identify the probable impact of mediation in the flipped classroom. The objectives of the study can be formulated into the following research questions.

### Research questions

1. Does mediation have any effect on the medical students’ writing in the flipped classroom?
2. Is there any difference between the writing performance of medical students in flipped and traditional classroom?
3. If any, which writing component(s) (content, organization, vocabulary, language use, and sentence mechanics) are more affected by mediation in flipped writing classroom of medical students?

According to the above research questions, the following hypotheses have been suggested:

1. Mediation has no effect on medical students’ writing in the flipped classroom.
2. There is no difference between the writing performance of medical students in flipped and traditional classroom.
3. None of the writing components is affected by mediation in flipped writing classroom of medical students.

## Method

### Design of the study

The present study investigated the effect of mediation in flipped writing classroom of medical university students. An experimental research design was used to compare the experimental group (a combination of mediation and flipped classroom) with the control group (traditional writing instruction).

### Participants

The study favored 47 medical students who enrolled in a 3-unit compulsory writing course as a prerequisite for the fulfillment of a degree at Shiraz University of Medical Sciences in the spring semester of 2019–2020. To control instruction effects, purposive sampling was implemented to select two writing classes that were instructed by the same writing instructor from among the six offered courses. The experimental and control groups consisted of 25 and 22 male and female students respectively.

To ensure that the participants were homogenous in their writing ability, they were required to complete a writing task before instruction. Later, the writings were marked and analyzed by an independent sample t-test. Since the *p*-value between the two writing groups was 0.408, it was concluded that the two writing groups were homogenous.

### Instruments

#### Medical students’ writing prompts

To assess the students’ writing level before instruction and make further comparisons possible, the students were asked to write a paragraph on childhood obesity as a serious problem in many countries at the beginning of the semester. They were asked to explain the main causes of this problem, elaborate on its effects, and suggest some possible solutions. With respect to the students’ major and the fact that they had already passed the nutrition course in the previous year, it was assumed that the students possessed the necessary background knowledge to complete the task appropriately.

The other implemented instrument was the students’ writings on the midterm exam. The students in both the experimental and control groups were asked to write on the topic of health promotion and whether they felt that it needed to be built into all the policies.

The midterm exam was given after 3 months (12 weeks) from the beginning of the semester. The reason for specifying a 12-week time interval can be attributed to the fact that a 6- to 8-week intervention has generally been considered appropriate for experimental studies (Chwo, Marek, & Wu, 2018). Besides, a 12-week time interval can minimize the novelty effect.

#### Writing grading rubric

The Analytic Rubric (Jacobs et al., 1981) was used to score the participants’ writing samples. The scale assesses writing ability using the traits of content, organization, language use, vocabulary, and mechanics. In this rubric, the content section assesses knowledgeable, substantive, and thorough development of the thesis statement. The organization part assesses the fluency of expression, clarity in the statement of ideas, support, organization of ideas, and sequencing and development of ideas. The vocabulary part looks at the sophisticated range, effective word choice, word form mastery, and appropriate register. The language use section is concerned with the use of effective complex construction, agreement, tense, number, and word order. The mechanics section deals with spelling, punctuation, capitalization, and paragraphing. The scores which are allocated to each trait are as follows: content, 25; organization, 25; language use, 25; vocabulary, 15; and mechanics, 10. The total mark was100 points.

#### Writing video contents used in the flipped classroom

According to the educational rules and regulations of Iranian medical universities, 30% of the writing instructions in all majors in medical universities in Iran could be flipped, so out of 17 weeks of writing instruction comprising of 34 90-min session writing lessons, 6 sessions were flipped using pre-recorded grammar lessons recorded professionally in the Virtual University of Medical Sciences. These lessons were uploaded to Navid (http://sumsnavid.vums.ac.ir/) which is a learning management system used by all medical universities across Iran. Accessing Navid requires the student’s ID number and the self-created password. The videos were uploaded and made accessible only to the students of the experimental group. To ensure that the students in the control group could not access the files on Navid, the download option was disabled. Furthermore, since the research design of the present study required the students to watch the videos prior to attending writing class, their viewing habits were monitored through Navid, and reminders were sent to those who had not watched the videos.

Note that all of the writing videos uploaded to Navid were recorded and edited professionally at Virtual University of Medical Sciences and approved by the advisory committee there. More information regarding the software used for making videos and the snapshots of the introductory video uploaded to Navid can be seen in Appendices 1 and 2. The syllabus given to the students of the experimental group at the beginning of the semester can be seen in Appendix 3 in Table 6.

#### Data collection procedure

To collect the data, the participants were divided into two groups as the control in which the traditional instruction was carried out and the experimental group in which the flipped classroom was conducted. The researchers explained the objectives and benefits of the study, and then, a consent form was distributed to seek the participants’ permission and tendency to be included in the study. Before an experiment, both groups were asked to write a paragraph. Then, the researchers started the experiment which lasted for six 90-min sessions in 4 weeks. The experimental group received flipped classroom, whereas the control group received traditional instruction. After that, the students were asked to write a paragraph again on a specific topic explained in the earlier section to collect the data after the treatment (posttest).

### Intervention

#### Experimental group treatment: mediation in the flipped classroom

For this study, students were required to have already watched the assigned videos completely before attending their writing class. They were asked to watch typically 20–30-min video lectures of instructions in six 90-min sessions covering a particular topic including different types of sentences in English, parallel structure, subject-verb agreement, noun clauses, adjective clauses, and adverbial clauses.

To ensure allocating equal class time for experimental and control groups, the time students spent watching clips related to each session was deducted from their class time, and the remaining was spent to do exercises. Thus, when attending the flipped classroom sessions, students were required to do class exercises (such as editing and rewriting paragraphs) provided by their writing instructor individually or in groups. In this way, the writing contents that were usually taught by the writing instructor in the classroom were covered by students themselves at home, and the assignments that were typically done at home were covered and supervised by the writing instructor in the classroom. A thorough sample of how one or two writing sessions proceeded is discussed in the following parts.

#### Second week/first mediation

Using modeling as one of the major scaffolding techniques in writing, the students were required (in pairs/groups) to study certain sentences in the prompt and identify certain features (Appendix 4).

Using teacher-student and student-student conferencing strategy, the students were asked to reflect on their tasks (connecting the sentences). The writing instructor offered further on the spot explanation and guided the students, if necessary (Le & Nguyen, 2010). The assistance was gradually withdrawn as the students internalized the patterns (Lipscomb, Swanson, & West, 2010).

While the students were reading and discussing the punctuation or grammatical points, the teacher explained and discussed the errors to the students. Errors like fragments, comma splices, run-ons, and stringy sentences were also extensively discussed. This scaffolding technique is called “offering explanation” and includes explicit teaching to develop the students’ understanding of declarative knowledge, conditional knowledge, and procedural knowledge (Roehler & Cantlon as cited in Dewi, 2013).

Finally, using talk-aloud modeling, the writing instructor showed the medical students the original model of the paragraph to explicitly explain, analyze, and discuss the genre, academic vocabulary used, and grammatical patterns applied in the model (Lange, 2002).

#### Fourth week/second mediation

To further explore and practice the video contents, the students were asked to read a paragraph about aspirin and not only use the strategies that they had been taught previously to make the paragraph more coherent, but also expand the paragraph by adding more information to and punctuate it appropriately. This scaffolding technique which is called “Joint Construction” is the strategy in which the writing instructor helps the students to construct a similar text within the given text. To give the students a better understanding of aspirin, a video was shown to them twice (https://www.uspharmacist.com/article/lowdose-aspirin), and they were asked to write down whatever information they thought might help them write a paragraph on aspirin using different types of sentences. Using this scaffolding technique, which is called “Contextualization,” the writing instructor provided sensory context by using pictures, manipulatives, film, or authentic objects to enhance the students’ understanding and made the concepts more relatable. The “schema-building strategy” was also used to discuss and brainstorm the topic in pairs or groups. This strategy can lead to the activation of the students’ schemata (Sun, 2014).

### Control group: traditional writing instruction

The students in the control group received conventional instruction consisting of teaching all the contents covered by the experimental group (the structure of the paragraph, process paragraph, compare and contrast paragraph). However, the grammatical lessons including sentence types, parallel structure, subject-verb agreement, adjective clauses, noun clauses, and adverbial clauses were fully covered in the classroom through six 90-min sessions, and the assignments were assigned as homework as the instructor was involved in teaching the materials in the classroom and there was limited time for doing assignments as classwork. In the following sessions, the writing instructor provided the answers to the students in the classroom and ensured they did not have any problems accordingly.

### Inter-rater reliability

Since the students’ pretests and posttests were analyzed and marked by two human raters (university lecturers), the inter-rater reliability was calculated (Neuendorf, 2002). Before marking the papers, the researchers discussed the writing rubric with the two raters comprehensively. To ensure the homogeneity of the scoring procedures, the raters were asked to mark two paragraphs using the rating rubric. Then, 47 assignments of the pretest and 47 assignments of the posttest were given to the raters, and a month deadline was determined for the writing rubric to be checked for each paper.

To assess the inter-rater reliability, Pearson product-moment correlation was calculated based on the average ratings on scores for the six individual components of each writing task. To this end, inter-rater reliability of the scores given by the two raters was examined. The reliability for total score was .678. The reliability for the components of content, organization, word choice, sentence structure, grammar, and mechanics was .681, .647, .743, .618, and .698 respectively. This is indicative of a high consistency between the two raters with respect to rating the participants’ knowledge of writing and its components. Moreover, the correlation coefficients were all significant indicating that both raters were significantly consistent in scoring the participants’ writing tasks.

### Data analysis

The data analysis was conducted in two phases. In the initial phase, the data were checked for the normal distribution assumption using a one-sample Kolmogorov-Smirnov test. According to the results of this test, the distribution of the scores was not normal, so non-parametric test including the Wilcoxon and Mann-Whitney test were used. In the next phase, descriptive statistics and the Wilcoxon signed-rank test were run to see whether mediation had any significant effect on different aspects of medical students’ writing in flipped classroom. Mann-Whitney test was also used to identify any significant difference between the writing performance of medical students in flipped and traditional classrooms. ANOVAs were also run to determine which writing component(s) (content, organization, vocabulary, language use, and sentence mechanics) were/was more affected by mediation in the flipped writing classroom. Caution was exercised to control the effect of pretests or computer difference between posttest and pretest mean scores (by Mann-Whitney test).

## Results

First, normality of the scores was examined to specify if parametric or non-parametric tests should be used. A one-sample Kolmogorov-Smirnov test was conducted to test normality of the scores. According to the results of the test (Appendix 6 in Tables 8, 9, 10, 11, 12, 13), the scores were not normally distributed, so non-parametric tests were used in the present study.

The results of the descriptive statistics (Table 1) show that the means for different components of the students’ writing and their writing performance before attending flipped classes were lower than their means after instruction.

**Table 1:** Paired sample descriptive statistics

Instruction			Mean	N	SD	Std. error mean
Flipped	Pair 1	Pre-content	10.6800	25	2.79464	0.55893
		Post-content	24.1200	25	1.42361	0.28472
	Pair 2	Pre-organization	10.8800	25	1.85562	0.37112
		Post-organization	24.2400	25	1.16476	0.23295
	Pair 3	Pre-vocabulary	6.9600	25	1.64520	0.32904
		Post-vocabulary	14.6000	25	2.25462	0.45092
	Pair 4	Pre-language use	11.1200	25	2.04776	0.40955
		Post-language use	23.7200	25	2.15097	0.43019
	Pair 5	Pre-sentence mechanics	6.0000	25	0.91287	0.18257
		Post-sentence mechanics	8.6000	25	0.70711	0.14142
	Pair 6	Pre-performance	45.6400	25	7.92612	1.58522
		Post-performance	95.2800	25	3.84621	0.76924

To see if the difference between pre- and posttest scores was statistically significant, Wilcoxon test was conducted. As the P values for all components of writing and the students’ writing performance were found to be lower than .05, it is concluded that these differences are statistically significant. This is indicative that the students showed a better writing performance after enrolling in the flipped classroom (see Table 2).

**Table 2:** Wilcoxon signed-rank test

Instruction		Pre-content Post-content	Pre-organization Post-organization	Pre-vocabulary Post-vocabulary	Pre-language use Post-language use	Post-sentence mechanics –pre-sentence mechanics	Post-performance –pre-performance
Flipped	Z	−4.405	−4.410	−4.388	−4.403	−4.432	−4.376
	Asymp. Sig. (2-tailed)	0.000	0.000	0.000	0.000	0.000	0.000

Table 3 illustrates the results for the posttest mean scores of the flipped and traditional groups. The mean of the writing components and the writing performance in the flipped group is higher than their counterparts in the traditional group.

**Table 3:** Descriptive statistics for the differences between posttest mean scores of flipped and traditional groups

	Instruction	Mean	N	Std. deviation
Post-content	Traditional	15.2273	22	2.84407
	Flip	24.1200	25	1.42361
Post-organization	Traditional	15.2273	22	3.19124
	Flip	24.2400	25	1.16476
Post-vocabulary	Traditional	10.3182	22	1.80967
	Flip	14.6000	25	2.25462
Post-language use	Traditional	14.6364	22	3.10982
	Flip	23.7200	25	2.15097
Post-sentence mechanics	Traditional	7.0909	22	0.92113
	Flip	8.6000	25	0.70711
Post-performance	Traditional	62.5000	22	9.20533
	Flip	95.2800	25	3.84621

To identify possible significant differences, Mann-Whitney test was conducted (see Table 4). Since the P value for all writing components and writing performance was smaller than 0.05, it was concluded that the students in the flipped classroom outperformed the students in the traditional class.

**Table 4:** Mann-Whitney test

	Post-content	Post-organization	Post-vocabulary	Post-language use	Post-sentence mechanics	Post-performance
Mann-Whitney U	0.000	0.000	16.000	12.000	62.500	0.000
Wilcoxon W	253.000	253.000	269.000	265.000	315.500	253.000
Z	−6.003	−5.980	−5.630	−5.675	−4.714	−5.871
Asymp. Sig. (2-tailed)	0.000	0.000	0.000	0.000	0.000	0.000

To see which writing component(s) (content, organization, vocabulary, language use, and sentence mechanics) were/was more affected by mediation in the flipped writing classroom, a multiple regression analysis was conducted (Appendix 5 in Table 7). The obtained results further pointed out that flipped classroom had the least and most impact on sentence mechanics and the students’ writing performance respectively.

## Discussion

### Mediation and writing performance

The results showed that mediation does have a significant effect on the writing performance of medical students when 30% of the writing instruction is flipped. The findings of the present study accord with the findings of Aziz Faraj (2015) who reported an improvement in the writing performance of Iraqi students after receiving scaffolding. The findings are also consistent with Gholami Pasand and Tahriri (2017) who concluded that university students majoring in English Language and Literature also gained beneficial results from scaffolding, especially from peer scaffolding in writing classrooms. Academic writing is closely attached to the learners’ productive skills rather than receptive ones (since the learners need to write and produce paragraphs and essays). Academic writing necessitates the students to put what they have previously learned and memorized into practice to productively create paragraphs and essays. So flipped classroom, which mostly allocates class time to practical tasks and exercises rather than theoretical delivery of the teaching material, would be a good alternative to traditional methods to writing instruction. This notion is consistent with the findings of the present study which showed that the only major difference in the control and experimental groups was the time spent on practical tasks and exercises.

### Flipped and traditional classrooms

In the flipped classroom, the instructor did not teach writing, and the students learnt all of the materials by watching prepared clips independently. The findings here also support the notion that self-regulated learning in a flipped classroom can enhance the students’ performance by saving more time for actual writing tasks and being involved in exercises compared to traditional classrooms in which the students do not have much opportunity to ask or answer questions (van Alten, Phielix, Janssen, & Kester, 2019). According to Schmidt, Wagener, Smeets, Keemink, and Van Der Molen (2015), the cognitive load associated with performing simultaneous tasks (i.e., teaching and class activity) in the classroom lowers the active construction of knowledge. Delivering instructions through student-controlled video lessons can compensate for this and increase information processing (Abeysekera & Dawson, 2015). Although Lai and Hwang (2016) believe that flipped classroom might be disadvantageous for students who have less learning autonomy in terms of self-regulated abilities, the result of the present study showed that since students are actively and collaboratively constructing meaning while doing the activities in the flipped classroom, knowledge is stored in their long-term memory, resulting in better performance. Using scaffolding techniques to gradually build upon students’ prior knowledge, teachers can actively guide the students throughout the class, the role which is absent when students independently work on their assignments after class.

Various writing mediations from modelling, asking students to apply knowledge to similar contexts, comparing and contrasting their writings with the original samples, dialoging, discussing and co-creating knowledge with their peers, etc. were used by the teachers in their classes in the present study. This is in line with a study conducted by Van den Bergh, Ros, and Beijaard (2014) who reported that the most ingredient of active learning is the provision of effective feedback on the behalf of the teacher. The authors concluded that there will be more room for this type of feedback when the class is flipped.

Contrary to Heyma et al. (2015) who reported that face-to-face classroom time was reduced by two-thirds in the flipped classroom, and, as a result, the students had a poor performance compared to the time they were traditionally instructed, we believe that since these students were secondary students, they still had not the ability to take responsibility for their learning, which might be in disagreement with studies reporting that flipped classroom can be equally effective in lower levels of education (see Lo & Hew, 2017 for instance).

### Class size effect and effectiveness of the activities

Although the present study’s small sample size might be considered as one of its limitations, the authors believe that the obtained results in the flipped writing classes can be attributed to the small number of students in these classes. This notion correlates with Schmidt et al. (2015), who stated that class size and lecture activities can have a significant role in the effectiveness of flipped classroom outcomes. The smaller class size can lead to more effective activities, which ultimately bring about better performance.

### Effects of flipped classroom on writing components

The findings also illustrate that in descending order “content,” “organization,” “language use,” “vocabulary,” and “mechanics” have been affected by flipped classroom. Despite the authors’ prior assumptions that “language use” would be the most affected writing component (because all of the multimedia contents used in the study were related to language use), the results indicated that “content” and “organization” were mostly affected by the mediation in flipped classrooms. This is in line with Aziz Faraj (2015) who concludes that when students’ role changes from passive learners to active ones in generating their meanings in writing, their focus shifts from “mechanics” to “content” and “organization”. Although it is generally assumed that lower number of skilled writers focus more on vocabulary and grammatical rules (August, 2002), the findings of the present study suggest that this can be changed when novice writers are offered the appropriate tools and are provided with the right scaffolding techniques.

The greater impact of flipped classroom on the writing components of “content” and “organization” can also be attributed to giving the students numerous editing practices that might have enhanced their self-monitoring skills. Self-assessment practice, according to Cresswell (2000), can engage learners in an evaluation process and encourage responsibility which can eventually lead to fewer content and organization errors in their final product.

The third writing component affected by mediation in flipped classroom was language use. According to Richards and Renandya (2008), “for the L2 students, many writing conventions will remain a mystery unless teachers can bring these forms and patterns of language use to conscious awareness” (p.321). Since meaning is only achieved via language, form is as equally important as content in the final product, so dual focus on content and language should be taken into consideration (Hyland, 2003; Ferris & Hedgcock, 2005).

The two least affected writing components in the present study were “vocabulary” and “mechanics”. In terms of “vocabulary,” one possible assumption is that since the medical students were given health-related topics from the very beginning of the writing course, and since they had already learned the medical and academic vocabularies and register in their clinical textbooks, they were already competent with their word choices, word forms, and register. Olinghouse and Wilson (2013) state that student writers vary in their vocabulary usage based on genre, mostly writing expository topics. In the present study, it is probable that since medical students had been already exposed to various high-level articles, they were able to produce a sophisticated range of vocabularies and appropriate register (Olson and Matuchniak, 2015). As for mechanics, it can be concluded that in a process-oriented approach, it is normally the “content” that is prioritized over grammatical accuracy and mechanics (Ferris, 2011, b).

## Conclusion and implications

First, by requiring students to study in advance and to take responsibility for their learning, flipped classroom can provide the opportunity for learners to actively construct knowledge by themselves rather than receiving the information passively in the classroom. Time wise, flipped classroom can cultivate interactive class time for teachers and enable them to invest in more academic practice, instead of asking students to spend a substantial amount of time each week outside the classroom doing homework independently. Second, scaffolding techniques can provide students with a more engaging and effective environment that helps them to have more social interaction while taking ownership of their learning. Through gradual scaffolds, writing instructors can pre-plan and use various instructional modes and means to bridge learning gaps at a certain point in the learning process. The main implication following our results is that flipped classroom should be implemented for higher education with packed syllabi.

## Data Availability

Raw data is available upon request.

## Appendix 1

**Table 5:** Information regarding the software used for making videos

Title	Time	Size (Meg)	Platform	Software
UNIT 1-lesson 1: clauses and phrases	5	9	LMS	Articulate Studio
UNIT 1-lesson 2: independent and dependent clauses	6	9	LMS	Articulate Studio
UNIT 1-lesson 3: sentence type	17	20	LMS	Articulate Studio
UNIT 2: parallel structure	26	30	LMS	Articulate Studio
UNIT 3: subject-verb agreement	19	20	LMS	Articulate Studio
UNIT 4: noun clauses	20	21	LMS	Articulate Studio
UNIT 5: adverbial clauses	20	21	LMS	Articulate Studio
UNIT 6: adjective clauses	30	25	LMS	Articulate Studio

## Appendix 3

**Table 6:** The writing syllabus given to the experimental group

Week	Content		Lesson delivery
1	Introduction/basics of a paragraph Students were asked to watch video content on “clauses and sentence types”		Verbal
2	1st session	Pre-writing and planning ideas	Verbal
	**2nd session**	**Mediation 1: modelling, teacher-student and student-student conferencing offering explicit explanation, internalization**	**Flipped**
3	Building paragraphs with examples (topic sentence, supporting sentences, developing sentences, etc.) Students were asked to watch video content on “Parallel Structure”		Verbal
4	1st session	Description, narration, classification	Verbal
	**2nd session**	**Mediation 2: modelling, joint construction, contextualization, schema-building**	**Flipped**
5	Process paragraph Students will be asked to watch video content on “Subject-verb Agreement”		Verbal
6	1st session	Process paragraph	Verbal
	**2nd session**	**Mediation 3: modelling and deconstruction, teacher-student and student-student conferencing**	**Flipped**
7	Compare and contrast paragraph Students will be asked to watch video content on “Noun Clauses”		Verbal
8	1st session	Compare and contrast paragraph	Verbal
	**2nd session**	**Mediation 4: modelling, bridging, prompting, negotiation**	**Flipped**
9	Argumentative paragraph Students will be asked to watch video content on “Adverbial Clauses”		Verbal
10	1st session	Argumentative paragraph	Verbal
	**2nd session**	**Mediation 5: modelling, bridging, prompting, negotiation**	**Flipped**
11	Cause and effect paragraph Students will be asked to watch video content on “Adjective Clauses”		Verbal
12	1st session	Cause and effect paragraph	Verbal
	**2nd session**	**Mediation 6: modelling, bridging, prompting, negotiation**	**Flipped**
13	Essay writing		Verbal
14	Essay writing		Verbal
15	Essay writing		Verbal
16	Essay writing		Verbal
17	Essay writing		Verbal

## Appendix 4

Model

Dangerous Allergies

1 Most people do not think of peanuts when they think of poisons. 2 Serious peanut allergies are putting an increasing number of individuals at risk for death. 3 Between 1997 and 2008, the peanut allergy rate among children in the United States nearly tripled. 4 The problem is causing great concern. 5 There are no signs that the allergy rate will decrease. 6 No one knows for certain why this increase is happening. 7 There are several theories. 8 Some believe that children are becoming more sensitive to allergens and cleaning products, and antibiotic medicines have made our environment too sanitary and this level of cleanliness, some say, leads to an underdeveloped immune system. 9 Another group of theorists believes that the problem lies in the way peanuts are prepared and this argument blames the roasting process for making peanuts more allergic. 10 Because severe allergies have risen at such an alarming rate it is likely that research into the causes of these allergies will grow so science may soon be able to explain why more and more children are threatened by such simple food.

*Taken and modified for this study from Longman Academic Series 4 Book*

1. Identify and briefly explain the individual clauses and sentences in the paragraph
2. Identify and explain the relationship between the sentences that are too short (simple sentences)
3. List what you need to know and/or know how to do to connect the simple sentences to make compound, complex, and compound-complex sentences
4. Connect the sentences with linking words and conjunctive adverbs (look at the handout)
5. Look at sentences 8 to 10, and identify the errors

## Appendix 5

**Table 7:** Multiple regression analysis

Source	Type III sum of squares	df	Mean square	F	Sig.	Partial eta squared
Sentence mechanics	26.650	1	26.650	40.219	.000	.472
Organization	950.555	1	950.555	173.583	.000	.794
Content	925.411	1	925.411	190.585	.000	.809
Vocabulary	214.546	1	214.546	50.608	.000	.529
Language use	965.571	1	965.571	138.320	.000	.755

## Appendix 6

**Table 8:** Test of normality (pre-content, pre-organization, and pre-vocabulary)

One-Sample Kolmogorov-Smirnov Test					
Instruction			Pre-content	Pre-organization	Pre-vocabulary
Traditional	N		22	22	22
	Normal parameters^a,b^	Mean	11.2727	11.6364	7.1818
		Std. deviation	2.31315	1.98915	1.29601
	Test statistic		.456	.398	.283
	Asymp. Sig. (2-tailed)		.000	.000	.000
Flipped	N		25	25	25
	Normal parameters^a,b^	Mean	10.6800	10.8800	6.9600
		Std. deviation	2.79464	1.85562	1.64520
	Test statistic		.334	.314	.280
	Asymp. Sig. (2-tailed)		.000	.000	.000

**Table 9:** Test of normality (pre-language use and pre-sentence mechanics)

One-sample Kolmogorov-Smirnov test				
Instruction			Pre-language use	Pre-sentence mechanics
Traditional	N		22	22
	Normal parameters^a,b^	Mean	12.2273	6.0000
		Std. deviation	2.20242	.43644
	Test statistic		.348	.409
	Asymp. Sig. (2-tailed)		.000	.000
Flipped	N		25	25
	Normal parameters^a,b^	Mean	11.1200	6.0000
		Std. deviation	2.04776	.91287
	Test statistic		.283	.340
	Asymp. Sig. (2-tailed)		.000	.000

**Table 10:** Normality test (post-content and post-organization)

One-sample Kolmogorov-Smirnov test				
Instruction			Post-content	Post-organization
Traditional	N		22	22
	Normal parameters^a,b^	Mean	15.2273	15.2273
		Std. deviation	2.84407	3.19124
	Test statistic		.243	.232
	Asymp. Sig. (2-tailed)		.001	.003
Flipped	N		25	25
	Normal parameters^a,b^	Mean	24.1200	24.2400
		Std. deviation	1.42361	1.16476
	Test statistic		.372	.343
	Asymp. Sig. (2-tailed)		.000	.000

**Table 11:** Normality test (post-vocabulary and post-language use)

One-sample Kolmogorov-Smirnov test				
Instruction			Post-vocabulary	Post-language use
Traditional	N		22	22
	Normal parameters^a,b^	Mean	10.3182	14.6364
		Std. deviation	1.80967	3.10982
	Test statistic		.233	.197
	Asymp. Sig. (2-tailed)		.003	.026
Flipped	N		25	25
	Normal parameters^a,b^	Mean	14.6000	23.7200
		Std. deviation	2.25462	2.15097
	Test Statistic		.390	.276
	Asymp. Sig. (2-tailed)		.000	.000

**Table 12:** Normality test (post-sentence mechanics and pre-performance)

One-sample Kolmogorov-Smirnov test				
Instruction			Post-sentence mechanics	Pre-performance
Traditional	N		22	22
	Normal parameters^a,b^	Mean	7.0909	48.3182
		Std. deviation	.92113	6.57112
	Test statistic		.202	.170
	Asymp. Sig. (2-tailed)		.020	.006
Flipped	N		25	25
	Normal parameters^a,b^	Mean	8.6000	45.6400
		Std. deviation	.70711	7.92612
	Test statistic		.274	.223
	Asymp. Sig. (2-tailed)		.000	.002

**Table 13:** Normality test (post-performance)

One-sample Kolmogorov-Smirnov test			
Instruction			Post-performance
Traditional	N		22
	Normal parameters^a,b^	Mean	62.5000
		Std. deviation	9.20533
	Test statistic		.142
	Asymp. Sig. (2-tailed)		.200
Flipped	N		25
	Normal parameters^a,b^	Mean	95.2800
		Std. deviation	3.84621
	Test statistic		.193
	Asymp. Sig. (2-tailed)		.017

## Abbreviations

L2: Second language
ESP: English for Specific Purposes
